# Antisense oligonucleotide-based targeting of Tau-tubulin kinase 1 prevents hippocampal accumulation of phosphorylated tau in PS19 tauopathy mice

**DOI:** 10.1186/s40478-023-01661-3

**Published:** 2023-10-19

**Authors:** Kayo Yukawa, Satomi Yamamoto-Mcguire, Louis Cafaro, Christine Hong, Fredrik Kamme, Tsuneya Ikezu, Seiko Ikezu

**Affiliations:** 1 Department of Pharmacology and Experimental Therapeutics, Boston University School of Medicine, Boston, MA 02118 USA; 2 Ionis Pharmaceuticals, Carlsbad, CA 92010 USA; 3 Department of Neuroscience, Mayo Clinic Florida, Jacksonville, FL 32224 USA; 4 Regenerative Science Graduate Program, Mayo Clinic Graduate School of Biomedical Sciences, Rochester, MN 55905 USA; 5 Mayo Clinic Alzheimer’s Disease Research Center, Rochester, MN 55905 USA; 6 Robert and Arlene Kogod Center on Aging, Mayo Clinic, Rochester, MN 55905 USA

**Keywords:** Alzheimer’s disease, Tau, TTBK1, Pathology, Neurodegenerative, Tauopathy, Phosphorylated tau protein

## Abstract

Tau tubulin kinase-1 (TTBK1), a neuron-specific tau kinase, is highly expressed in the entorhinal cortex and hippocampal regions, where early tau pathology evolves in Alzheimer’s disease (AD). The protein expression level of TTBK1 is elevated in the cortex brain tissues with AD patients compared to the control subjects. We therefore hypothesized that antisense oligonucleotide (ASO) based targeting *Ttbk1* could prevent the accumulation of phosphorylated tau, thereby delaying the development of tau pathology in AD. Here we show that in vivo administration of ASO targeting mouse *Ttbk1* (ASO-*Ttbk1*) specifically suppressed the expression of *Ttbk1* without affecting *Ttbk2* expression in the temporal cortex of PS19 tau transgenic mice. Central administration of ASO-*Ttbk1* in PS19 mice significantly reduced the expression level of representative phosphor-tau epitopes relevant to AD at 8 weeks post-dose, including pT231, pT181, and pS396 in the sarkosyl soluble and insoluble fractions isolated from hippocampal tissues as determined by ELISA and pS422 in soluble fractions as determined by western blotting. Immunofluorescence demonstrated that ASO-*Ttbk1* significantly reduced pS422 phosphorylated tau intensity in mossy fibers region of the dentate gyrus in PS19 mice. RNA-sequence analysis of the temporal cortex tissue revealed significant enrichment of interferon-gamma and complement pathways and increased expression of antigen presenting molecules (Cd86, Cd74, and H2-Aa) in PS19 mice treated with ASO-*Ttbk1*, suggesting its potential effect on microglial phenotype although neurotoxic effect was absent. These data suggest that TTBK1 is an attractive therapeutic target to suppress TTBK1 without compromising TTBK2 expression and pathological tau phosphorylation in the early stages of AD.

**Supplementary Information:**

The online version contains supplementary material available at 10.1186/s40478-023-01661-3.

## Introduction

Accumulations of abnormally phosphorylated tau (p-tau) are a pathological hallmark of Alzheimer’s disease (AD) and commonly associated with synaptic loss and neuronal damage [1]. Tau pathology in AD shows a hierarchical progression, which starts from the entorhinal cortex layer II (ECII) and then propagates to the hippocampal formation [2, 3]. Tau tubulin kinase-1 (TTBK1) is a member of a novel tau kinase family (TTBK1 and TTBK2) and specifically expressed in brains, more specifically in neurons [4]. Other groups and we recently demonstrated abundant TTBK1 protein and mRNA expression in the EC and CA1 in human brains [5, 6]. In addition, its expression is increased in the cortex tissue of AD patients’ brains as compared to the control group [7]. TTBK1 directly phosphorylates tau at one of AT8 recognition site (pSer^202^/pThr^205^) and pS422 (pSer^422^) [4, 8], which is characterized as a pre-tangle marker [9]. Eli Lilly’s group previously reported an unbiased screening of protein kinases that phosphorylate tau at AD-associated sites (Ser396/404, Thr235, Thr231, and Ser202) [10]. Their results identified TTBK1 as one of the top kinases phosphorylating tau at Ser202 and Thr231, which appears in early tau pathology in AD [10–12]. Genetic variations of *TTBK1 gene* are associated with late-onset AD in two cohorts of Chinese and Spanish populations [13, 14], further validating the importance of *TTBK1 gene* in the development of tau pathology in AD. Thus, suppressing initial pathological tau accumulation in the EC and their spread to the hippocampal region by targeting TTBK1 could halt AD progression at the prodromal stage. In this study, we investigated how suppression of *Ttbk1* expression by antisense oligonucleotide (ASO) targeting mouse *Ttbk1* (ASO-*Ttbk1*) affects the tau pathology development in PS19 mice.

## Materials and methods

### Animals

Breeding pairs of Tg (Prnp-MAPT*P301S) PS19Vle/J (PS19) mice were purchased from Jackson laboratory (Cat # 008169), and resulting progenies were maintained on a B6; C3 background. All non-transgenics used were littermates of PS19 mice. Mice had access to food and water ad libitum and were housed on a 12-h light: dark cycle. All animal procedures followed the guidelines of the National Institutes of Health Guide for the Care and Use of Laboratory Animals and were approved by Boston University Institutional Animal Care and Use Committee (IACUC).

### Antisense oligonucleotides (ASOs)

Mouse-specific ASOs, designed and provided by Ionis Pharmaceuticals, Inc., were 5-10-5 MOE gapmers, having a central gap region of ten phosphorothioate-linked deoxyribonucleotides flanked by five MOE-modified ribonucleotides on each of the 5′ and 3′ ends of the gap region. ASO sequences were as follows: *ASO-control*, CCTATAGGACTATCCAGGAA; *ASO-Ttbk1#1*, GCATATTTTTCCACTAGCCA; *ASO-Ttbk1#2*, GCGGCTTTTTTACCAACTTA.

### Intracerebroventricular (ICV) injection of ASOs

ASOs of *Ttbk1-* and control- were diluted in DPBS without Ca^2+^ and Mg^2+^ (Gibco, Cat # 14190144) and administered via intracerebroventricular (ICV) injection at a dose of 700 μg (in 10 μL), as previously described [15]. In brief, 6-month-age PS19 mice were positioned into a stereotaxic apparatus (Kopf) under anesthesia by continuous isoflurane inhalation. First, the skull was exposed, and then a small burr hole was drilled at the proper coordinates: anteroposterior, + 0.3 mm; mediolateral, + 1.0 mm; and dorsoventral, − 3.0 mm relative to the bregma (right hemisphere). Three minutes after the needle (Hamilton, Cat # 7758-04) connected to a 10 μL syringe (Hamilton, Cat # 7653-01) was placed into the proper coordinates, a total of 700 μg ASO diluted in 10 μL PBS was delivered at an infusion rate of 0.5 μL/s using an injection pump (World Precision Instruments). The mouse was allowed to recover in a temperature-controlled environment. Two out of 16 ASO-Ttbk1#1 treated mice and one out of 8 control treated mouse suddenly died 2–4 weeks after ICV administration, which did not show any statistical significance. Since the median survival of PS19 is already known to be 9 months [16], we considered these deaths that occurred at 7 months to be spontaneous. Following the surgery, weight, grooming activity, and home cage activity were recorded for up to 10 days according to IACUC guidelines.

### Euthanasia and tissue dissection

Eight weeks after ASOs treatment, mice were euthanized by transcardiac perfusion with ice-cold PBS. Brains were immediately removed and separated into the right and left hemispheres for the tissue collection. The right hemispheres were dissected into the region (frontal cortex, temporal cortex, and hippocampus) on a filter paper and frozen at − 80 °C. The left hemispheres were fixed with 4% paraformaldehyde/PBS for 16 h, then 30% sucrose/PBS over 3–5 days.

### mRNA isolation and real-time qPCR analysis

The temporal cortex was homogenized with Trizol reagent (Invitrogen, Cat # 15596026) and purified with RNeasy mini kit (Qiagen, Cat # 74104) following the manufacture protocol. cDNA was prepared from mRNA with SuperScript IV VILO Master Mix (Invitrogen, Cat # 11756050). cDNA samples (40 ng RNA templates), in quadruplicate, were measured for target genes using TaqMan fast advanced MasterMix(Applied Biosystems, Cat # 44-445-57) on 7500 HT PCR systems (Applied Biosystems). Quantification of target genes (*Ttbk1* and *Ttbk2*) was determined by the comparative Ct (ΔΔCt) method normalized with *Gapdh* as an endogenous control. The following qPCR probes were purchased from Thermo Fisher Scientific; Ttbk1 ((TaqMan probes Thermo Fisher Scientific, Mm01269698_m), Ttbk2 (Mm00453709_m1), Tmem119 (Mm00525305_m1), Tgfbr1 (Mm00436964_m1), Tyrobp (Mm00449152_m1), Itgax (Mm00498701_m1), Trem2 (Mm04209424_g1), and Lgals3 (Mm00802901_m1).

### Biochemical sequential extraction of sarkosyl soluble and insoluble fractions from mouse brain tissues

The hippocampal brain tissues were sequentially centrifuged for the enrichment of tau oligomer and fibrils according to the protocol previously reported [8, 17]. Briefly, each hippocampal tissue was homogenized in TBS buffer supplemented with PhosStop (Roche, Cat # 11699695001) and cOmplete (Roche, Cat # 04693116001). The homogenate was centrifuged at 150,000 g for 15 min at 4 °C, and the supernatant was collected as soluble (S1) fractions. Next, the pellet was homogenized in PHF-1 buffer, salt/sucrose buffer supplemented with PhoStop and cComplete and centrifuged as above. The resultant pellets were discarded, and the supernatants were incubated with sarkosyl (Sigma, Cat # 61747, 1% final concentration) for 1 h at 37 °C. Subsequently, the mixtures were spun in at 150,000 g for 30 min at 4 °C. The supernatants were collected as S2 fractions, while the pellets were collected as P2 fractions.

### Western blotting and ELISA

The protein concentration in each fraction was measured by BCA Protein Assay Kit (Pierce, Cat # 23225). As for pS422 detection, the S1 sample was denatured in 4 × Laemmli Sample Buffer (Bio-Rad, Cat # 161-0747) by boiling for 10 min at 95 °C. Proteins were loaded into a 4–20% tris–glycine gel (Bio-Rad, Cat # 456-1096) and separated by SDS-PAGE (Bio-Rad, Cat #1610732) at 110 V for 80 min. Gels were transferred to an IBlot nitrocellulose membrane (Invitrogen, Cat # IB301001), blocked with TBST blocking buffer (LI-COR Biosciences, Cat # 927-80001) for 1 h. The membrane was probed with primary pS422 antibody (Abcam, Cat # ab79415, 1:1000 dilution) and total tau (tau 5, Abcam, Cat # ab80579) in antibody dilution buffer (1:1 TBST blocking buffer and 1X TBST) overnight at 4 °C. After primary antibody staining, the blot was then washed in triplicate with TBST and incubated for 1 h with secondary anti-Rabbit-HRP antibody (Cell Signaling Technology, Cat # 7074S, 1:5000 dilution) or anti-Mouse-HRP antibody (Cell Signaling Technology, Cat # 7076S, 1:5000 dilution) . After a final triplicate wash with TBST, the blot was incubated with chemiluminescent HRP Substrate (Millipore Sigma, Cat # WBKLS0100 ) and then visualized with C300 digital chemiluminescent imager (Azure Biosystems). The optical band intensity was measured by Image Studio Lite (LI-COR Biosciences). The level of other phosphor-tau proteins (pT181, pT231, and pS396) in S1, S2 and P2 fractions isolated from the hippocampal tissue was measured by ELISA (Invitrogen, Cat # KHO0631, KHB8051 and KHB7031 resepctively) following the manufacture protocol. The protein level of TTBK1 in S1 fraction isolated from the hippocampal tissue was measured by ELISA (MyBioSource, Cat # MBS9341780) following the manufacture protocol.

### Immunofluorescence and quantification by Imaris

The fixed brains were cut coronally in 30 μM thickness using a cryostat. The sections were processed by antigen retrieval with Tris–EDTA (pH9.0), permeabilized in 0.5% Triton/PBS, and blocked in 10% Donkey serum (Sigma-Aldrich, Cat # D9663-10ML), 1% BSA, and 0.1% Tween20 in PBS. The section was incubated with pS422 (Abcam, 1: 500) at 4 °C overnight. Sections were washed and incubated in secondary antibody (AlexaFluoro-568 Donkey anti-Rabbit, ThermoFisher Scientific, Cat #  A10042, 1: 1000) for 3 h at room temperature. All images were captured on Leica TCS SP8 systems with X20 objective using Z-stacks (3 μm). These images are constructed to a 3D image in Imaris 9.5, 64-bit version (Bitplane AG). The quantification of pS422 was performed on the “surface” module. Because of lack of the segmentation, the mossy fiber region was manually identified, and mean of the intensity of pS422 in the region was calculated. As for the CA1, after 3D constructing using Imaris, the volume stained with pS422 antibody was calculated and normalized by CA1 region volume.

### RNA quality control and RNA seq

RNA concentration and purity of the temporal cortex were confirmed by Bioanalyzer 2100 (Agilent Technologies). All samples had a RIN between 9.4 and 10.0, and the average RIN was 9.70. These RNAs were provided to UChicago genomic core (RRID: SCR_019196) for next generation sequencing  using NovaSeq 6000 (Illumina). The reads were mapped to the GRCm38 reference transcriptome using STAR, version 2.6.1d. The quality of raw reads, as well as the results of STAR mapping, are generated using FastQC and MultiQC. Next, raw reads were mapped to the GRCm38 reference transcriptome using Salmon, version 1.5.2. The quality of raw reads and the results of Salmon mapping were summarized in this report. After read mapping with Salmon, (https://bioconductor.org/packages/release/bioc/html/tximport.html) was used to read Salmon outputs into the R environment (Additional file 1: Fig. S7 R Session information). Annotation data from Gencode vM25 was used to summarize data from transcript-level to gene-level. We first removed the non-protein coding genes. This reduced the number of genes from 54,418 to 21,828. Next, genes with less than 1 count per million (CPM) in at least 3 or more samples were filtered out. This exclusion reduced the number of genes from 21,828 to 13,758.

### Statistical analysis

All data are presented as means ± SEM. Comparisons between the two groups were made by unpaired Student’s t-test. Multiple comparisons were performed by either one- or two-way ANOVA, followed by Turkey’s. Statistical analysis was performed using Prism 9.0 (GraphPad software). A statistically significant difference was assumed at *p* < 0.05.

## Results

### ASO-*Ttbk1* selectively suppresses *Ttbk1* expression in the temporal cortex of PS19 mice

**Fig. 1:**
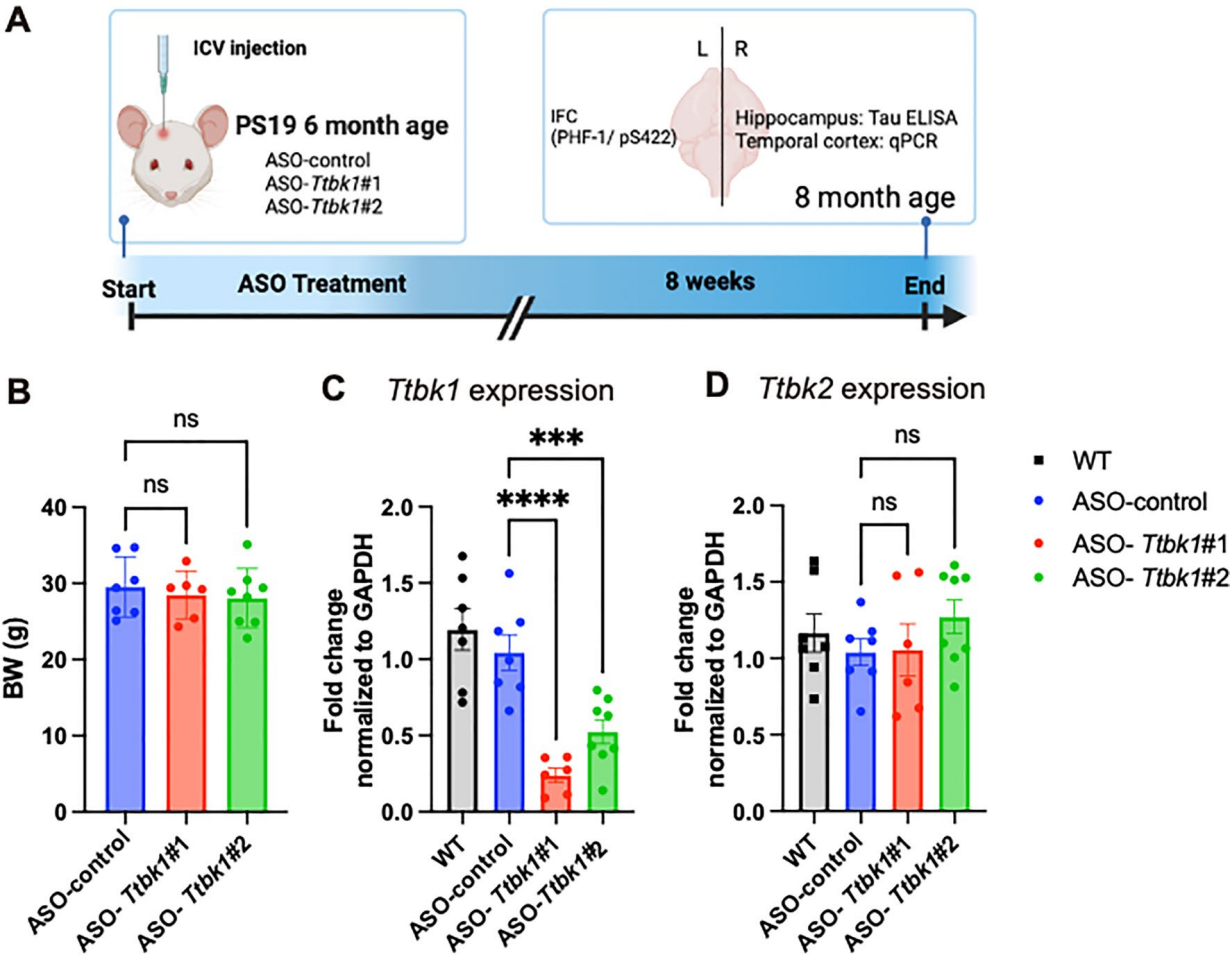
*Ttbk1* ASOs selectively suppress *Ttbk1* expression in the temporal cortex of PS19. **A** The scheme of the experimental design for ASO-mouse *Ttbk1* injection in PS19 mice. PS19 mice were injected with ASO-*Ttbk1* #1 or #2, or ASO-control at 6 month of age. Mice are euthanized 8 weeks after the injection for qPCR, ELISA, and immunohistochemistry. **B** Body weight measurement at the end point. **C**, **D** qPCR analysis of mRNA level of temporal brain tissue for *Ttbk1* (**C**) and *Ttbk2* (**D**). Reduced mRNA level of *Ttbk1* but not *Ttbk2* was observed by ASO-*Ttbk1* treatment. **** denotes < 0.0001 and *** denotes < 0.001 compared to ASO-control treated group as determined by one-way ANOVA and Dunnett’s multiple comparison (WT saline treated n = 6, ASO-control n = 7; ASO-*Ttbk1* #1 n = 6; ASO-*Ttbk1* #2 n = 8 mice)

Recently reported TTBK1 inhibitors target not only TTBK1 but also TTBK2, which loss of function mutation is known to lead to spinocerebellar ataxia 11 due to the similarity of catalytic domains between these two tau-kinases. To overcome this cross-reactivity, we employed ASOs which specifically target mouse TTBK1 but not TTBK2 designed and provided by Ionis Pharmaceuticals. First, we tested whether in vivo administration of two ASOs targeting *Ttbk1* (#1 and #2) in mouse brains would alter mouse *Ttbk1* mRNA expression. Six-month-old PS19 mice, which express human tau carrying the P301S mutation, were administered with 700 μg of ASO-*Ttbk1* or a control ASO (ASO-control) by intracerebroventricular (ICV) injection and euthanized for biochemical and immunohistological analysis 8 weeks after the ASO administration (Fig. 1A). As PS19 mice are known to form neurofibrillary tangles in the hippocampus and amygdala at 6-months-age [16], we hypothesized that administration of ASO-*Ttbk1* at this age would block phosphorylated tau accumulation in PS19 mice. There was no difference in body weight among the groups when they were euthanized (Fig. 1B). In order to determine the effect of ASO-*Ttbk1*, mRNA was isolated from the temporal cortex and assessed its expression level for *Ttbk1* and *Ttbk2*. Both ASO-*Ttbk1* #1 and #2 groups showed a significant reduction in *Ttbk1* expression compared to the ASO-control group, while *Ttbk2* expression was not affected (Fig. 1C, D). These results showed that in vivo administration of ASO- *Ttbk1* selectively inhibited *Ttbk1* expression in the temporal cortex in PS19 mice.

### ASO-*Ttbk1* reduces pS422 tau in the soluble fraction of the hippocampal tissue in PS19 mice

**Fig. 2:**
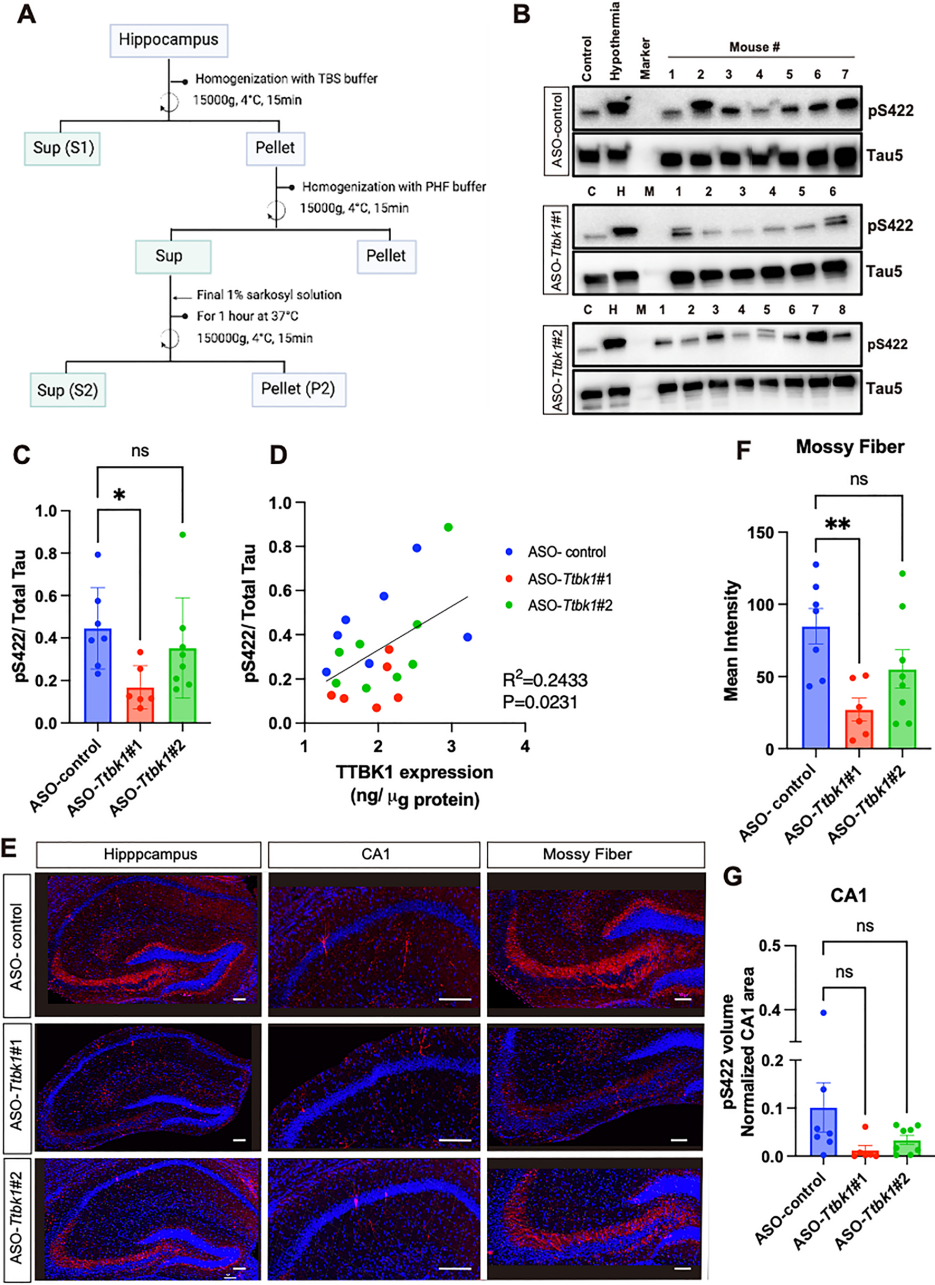
*Ttbk1 *ASOs suppresses pS422 tau level in PS19 mice. **A** Protein extraction scheme. **B** Western blot results against pS422 and total tau (tau5) antibodies with soluble fraction S1 isolated from the hippocampal tissue 8 weeks after the injection of ASO-*Ttbk1* or -control. C: WT mouse sample H: WT mouse samples under hypothermia to induce tau phosphorylation as a positive control, M: Marker, 1–8: Samples from PS19 mice injected with ASO-control, ASO-*Ttbk1*#1, or ASO-*Ttbk1*#2. **C** Protein expression levels of pS422 normalized by total tau**.** All membranes were always loaded with a constant amount of the same positive control (PC) at the same time, and all bands of pS422 and tau5 were quantified as a ratio to PC. One-way ANOVA and Dunnett’s multiple comparison. * denotes < 0.05. **D** Association analysis of pS422 level normalized by total tau and TTBK1 protein level in S1 fraction of the hippocampal tissues measured by ELISA. The GraphPad was used to confirm the Gaussian distribution, and then simple liner regression analysis was performed. **E** Representative immunofluorescence image of phosphorylated tau (pS422) in the hippocampal region in PS19 mice injected with ASO-control, ASO-*Ttbk1*#1, or ASO-*Ttbk1*#2 at 8 weeks post injection. pS422 (red) and DAPI (blue) immunostaining in the CA1 and mossy fiber. Scale bar = 100 μm. **F** Mean intensity measurement of pS422 in the mossy fiber region. **p < 0.01 compared to ASO-control treatment group as determined by One-way ANOVA and Dunnett’s multiple comparison. **G** Quantification of pS422 positive neuronal volume normalized by total CA1 volume (ASO-control n = 7; ASO-*Ttbk1* #1 n = 6; ASO- *Ttbk1* #2 n = 8 mice)

Next, we investigated the effect of ASO-*Ttbk1* on the level of pS422, previously identified as the direct phosphorylation site by TTBK1 [4, 8, 18]. It has been reported that the soluble fraction of tau isolated from brain tissue homogenates from AD patients enriches pS422 in early Braak stages [19]. Thus, we isolated the soluble fractions (S1) from the brain homogenate of the hippocampal tissues in PS19 mice at 8 weeks after ASO injections and WT mice (Fig. 2A). The brain samples from WT mice, which underwent hypothermia to induce phosphorylation of the pS422 site [20], were used as a positive control for the normalization between different western blot analyses (Fig. 2B, Additional file 1: Fig. S2A, B). ASO-*Ttbk1* #1 treated group, compared to the ASO-control group, showed significant reduction in pS422 normalized by total tau (tau5) (Fig. 2C). On the other hand, ASO-*Ttbk1*#2 treated group did not show a predominant decrease in pS422 with high individual variability. We considered this representing the same trend as the result of weaker suppression of *Ttbk1*mRNA by ASO-*Ttbk1* #2 compared to #1 (Fig. 1C). Furthermore, we found a significant association between pS422 and TTBK1 protein level (*p* = 0.0231) (Fig. 2D). Moreover, immunohistochemistry against pS422 (for pSer^422^ tau) was performed in the hippocampal field to determine the therapeutic efficacy of ASO-*Ttbk1*. We examined mossy fiber and CA1 regions where phosphorylated tau (p-tau) was detected in the early stage of tauopathy without the cortical atrophy in PS19 mice [21]. ASO-*Ttbk1* #1 treated group showed a significantly suppressed intensity of pS422 (*p* < 0.01) in mossy fiber compared to ASO-control treated group (Fig. 2E, F). The volume of pS422 in the CA1 region also showed a decreasing trend in the ASO-*Ttbk1* treated group, although it was not significant (Fig. 2G). Taken together, these results demonstrated that ASO-*Ttbk1* administration at 6 months of age efficiently suppressed the subsequent p-tau formation at pS422 site in the hippocampus in PS 19 mice, 8 weeks after the administration of ASOs.

### ASO-*Ttbk1* reduces pS396-, pT231-, and pT181-tau levels in the hippocampal tissue in PS19 mice

**Fig. 3:**
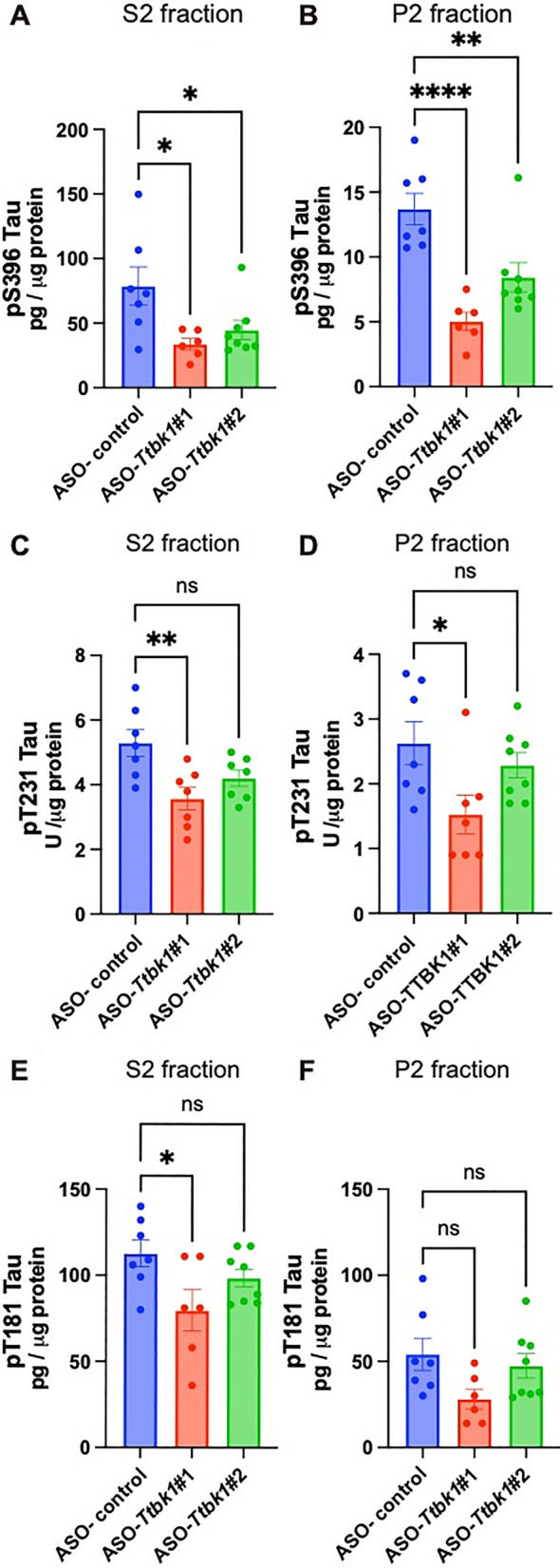
Phosphorylated tau level in PS19 mice treated with ASO targeting *Ttbk1*. Protein expression levels determined by ELISA for p-tau for epitope pS396 (**A**, **B**), pT231 (**C**, **D**) and pT181 (**E**, **F**), and total tau in sarkosyl soluble (S2; **A**, **C**, **E**) and insoluble (P2; **B**, **D**, **F**) fractions isolated from the hippocampal brain tissue in PS19 mice after ASO treatment. * denotes < 0.05, ** < 0.01, *** < 0.00, **** < 0.0005 compared to ASO-control treated group as determined by one-way ANOVA and Dunnett’s multiple comparison (ASO-control n = 7; ASO-*Ttbk1* #1 n = 6; ASO-*Ttbk1* #2 n = 8 mice)

Next, we evaluated the effect of ASO-*Ttbk1* on the other p-tau levels in the hippocampal tissue in PS19 mice. Hippocampal tissue from ASO-treated PS19 mice was homogenized and then sequentially centrifuged and separated into soluble (S1), sarkosyl-soluble (S2, tau oligomer) and -insoluble (P2, tau fibril) fractions (Fig. 2A). We measured the level of p-tau in each fraction by ELISA [22]. We found that ASO-*Ttbk1*#1 significantly reduced pS396, one of the phosphor epitopes in paired helical filaments (PHF) in S1 (*p* = 0.0332), S2 (*p* = 0.0138) and P2 (*p* < 0.0001) fractions (Additional file 1: Fig. S1A, Fig. 3A, B), pT231in S2 (*p* = 0.0043) and P2 fractions (*p* = 0.0225) and pT181 tau in S2 fraction (*p* = 0.0246), both of which are early AD biomarkers in cerebrospinal fluid [11, 12, 23, 24], compared to ASO-control group (Fig. 3C–F). ASO-*Ttbk1*#2 showed a significant reduction only in pS396 in S2 (*p* = 0.0453) and P2 fraction (*p* = 0.0045) (Fig. 3A and B)*,* parallel to its lesser silencing effect on *Ttbk1* expression compared to ASO-*Ttbk1*#1 (Fig. 1C). There was no change in pT231 and pT181 in S1 fraction or total tau by both ASO-*Ttbk1* (Additional file 1: Fig. S1B–D). Next, we performed a correlation analysis of p-tau and the protein expression level of TTBK1 in the S1 fraction of the hippocampal tissue determined by ELISA. We found that the pT231 level showed the strongest correlation with TTBK1 protein expression in the hippocampus, suggesting that TTBK1 may be involved in early pre-tangle formation (Additional file 2: Table S1) [11, 12]. These results suggest that in vivo injection of ASO-*Ttbk1* successfully suppressed p-tau formation at multiple sites relevant to AD pathology in the hippocampus in PS19 mice, validating its inhibitory effect on the pre-tangle formation in early-stage AD.

### TTBK1 KD induces immune activation via upregulation of antigen presentation, complement and interferon-γ pathway

**Fig. 4:**
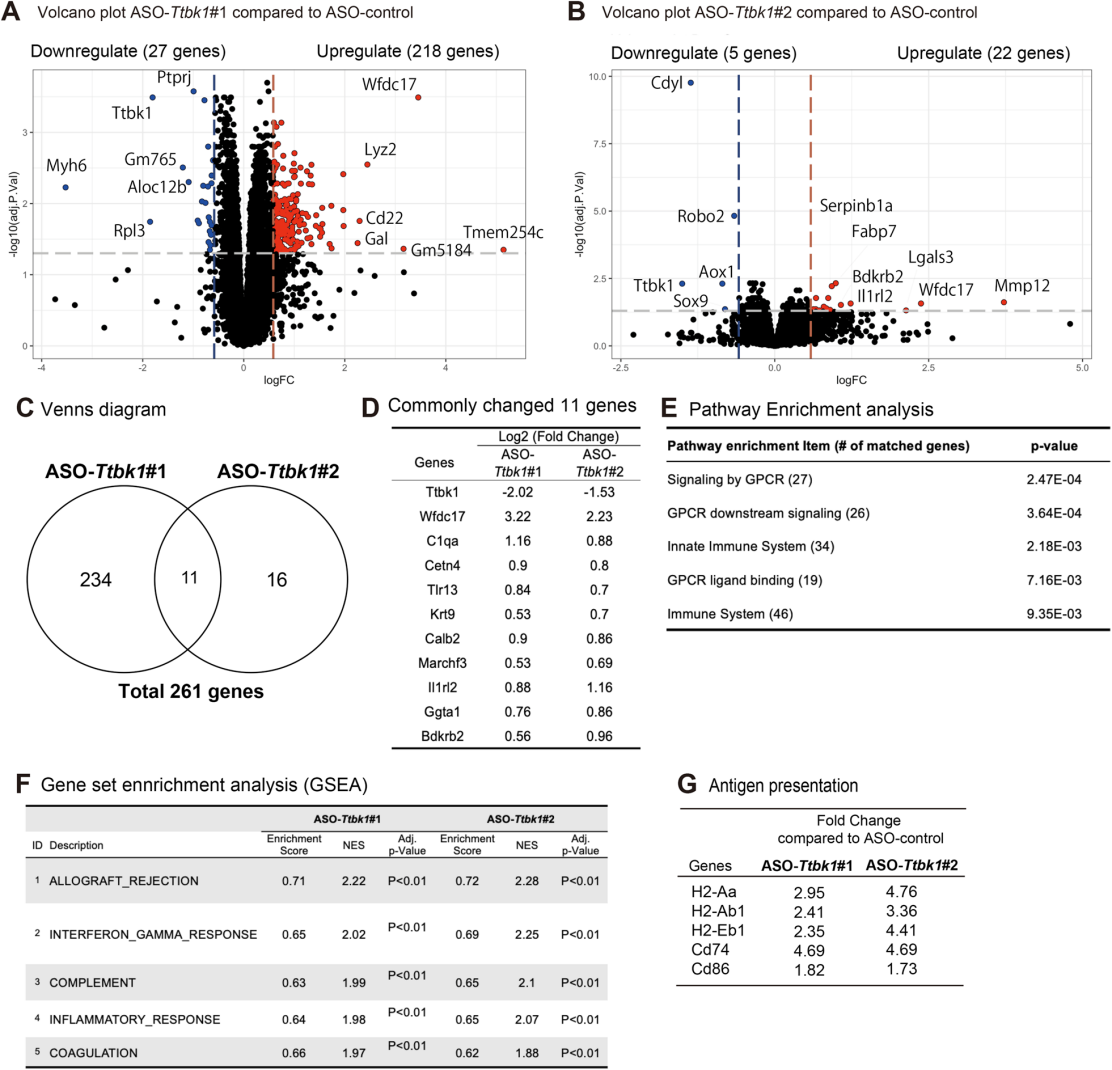
RNAseq analysis of the temporal cortex from PS19 mice treated with ASO-*Ttbk1*. **A**, **B** Volcano plot for the comparison between ASO-control and ASO-*Ttbk1* treated group. The horizontal axis shows log2 (fold change) and the vertical axis means log10 (adjusted p-value). Cutoff values were set at ± 1.5 for FC and 0.05 for adj. *p* value. **C** Genes significantly altered by ASO-*Ttbk1* treatment compared to ASO-control in the venn diagram. Cutoff values were set at ± 1.5 for FC and 0.05 for adj. *p* value. **D** List of 11 genes commonly and significantly altered by ASO-*Ttbk1*#1 or #2 treatment. The numbers represent the Log2 (Fold change). **E** Results of pathway enrichment analysis by Reactome for genes significantly altered by ASO-*Ttbk1*#1 treatment. **F** Top list of gene set enrichment analysis (GSEA) by ASO-*Ttbk1*#1 and #2 treatment. NES; Normalized enrichment Score. All Adj. *p* values were below 0.01. **G** Fold change in genes involved in antigen presentation by ASO-*Ttbk1*#1 and #2 treatment

To determine the transcriptomic change mediated by ASO-*Ttbk1*, we performed bulk RNA sequence analysis of isolated temporal cortical tissues, which is one of the brain regions where TTBK1 is highly expressed [2, 3]. 13,578 genes were extracted and used for the subsequent analysis. Principal-component analysis (PCA) showed that the ASO-control group sample was cleanly separated from ASO-*Ttbk1*#1 and #2 along with PC1 (Additional file 1: Fig. S3A). Genes with fold change more than 1.4-fold and adjusted p-value less than 0.05 were extracted from ASO-*Ttbk1*#1 or #2 treated group compared to ASO-control (Fig. 4A, B). The results showed that 245 genes in the ASO-*Ttbk1*#1 group and 27 genes in the ASO-*Ttbk1*#2 group were significantly differentially expressed compared to ASO-control. We observed fewer numbers of differentially expressed genes (DEG) in the ASO-*Ttbk1*#2 group, reflecting the lesser *Ttbk1* KD efficacy by ASO-*Ttbk1* #2 compared to ASO-*Ttbk1*#1 as shown in Fig. 1C. There were 11 common DEG among two groups (Fig. 4C), including *Ttbk1* and C1qa (Fig. 4D). Top 5 biological pathways identified by the Reactome analysis of DEG include G protein coupled receptor (GPCR) and the innate immune system (Fig. 4E). Next, we performed Gene Ontology (GO) enrichment analysis of DEG using g:Profiler (Fig. S4A–C). The classification of the extracted GO data revealed the highest number of GOs related to biological process. Furthermore, the top three GO terms identified immune response, regulation of multicellular organismal process, and defense response, confirming the enrichment of immune signaling (Additional file 1: Fig. S4C). Gene Set Enrichment Analysis (GSEA), which incorporates all analyzed genes, not just DEG, was used to confirm the accuracy of the extracted GO (Fig. 4F and Additional file 1: Fig. S5). The GSEA analysis showed a predominant positive shift in normalized enrichment score (NES) for allograft rejection, interferon-gamma response, and complement, suggesting that both ASO-*Ttbk1* may induce immune activation in PS19 mice. Allograft rejection and interferon-gamma response pathways share genes related to major histocompatibility class II molecules for antigen presentation, such as H2-Aa, H2-Ab1, H2-Eb-1, CD74, and Cd86 (Fig. 4G and Additional file 2: Table S2). In addition, Enricher analysis revealed that microglia or myeloid cell related DEG were most enriched by ASO-*Ttbk1* #1 treatment (Additional file 2: Table S3). We therefore evaluated the expression level of microglia-related genes by qRT-PCR, which showed significant increase in neurodegenerative microglia markers, *Trem2, Lgals3, Tyrobp*, and *Itgax* [25] and a homeostatic microglial marker, *Tmem119,* but not *Tgfbr1* in ASO-*Ttbk1* #1 or #2 group (Additional file 1: Fig. S6). This suggests a mixed response of microglia for both disease-associated and homeostatic phenotypes by ASO-*Ttbk1 *treatment at tissue level. Together, these data suggest that knockdown of *Ttbk1* induces immune activation, which is reflected in the upregulation of antigen presentation, complement and interferon-γ pathways in the temporal cortex in the treated PS19 mice.

## Discussion

In this study, we have shown that in vivo central administration of ASO-*Ttbk1* selectively downregulates *Ttbk1* expression in the temporal cortex tissue in PS19 mice without affecting *Ttbk2*. The catalytic domain of *Ttbk2* is highly homologous to *Ttbk1* and loss of function mutations of human *TTBK2* is genetically linked to spinocerebellar ataxia type 11 [26]. ASO-*Ttbk1* significantly suppressed p-tau (pT231, pT181 and pS396) level in the hippocampal tissue determined by ELISA and pS422 intensity in mossy fibers in the dentate gyrus.

In vivo tau hyperphosphorylation results from multiple tau kinase activities [27]. Therefore, targeting single tau kinase may not be enough to suppress tau phosphorylation. The drug development targeting glycogen synthase kinase 3B (GSK3B) and CDK5 kinases have been unsuccessful due to their ubiquitous expression and unwanted off-target effects. Since TTBK1 is expressed specifically in the CNS and has multifaceted effects, including the activation of CDK5 kinase, we can also target CNS specific CDK5 [7]. Although recent studies show the development of compounds targeting TTBK1, all lead compounds are known to cross-react with TTBK2 due to the high similarity in the catalytic domain of TTBK1 and TTBK2 [28–35]. Since loss-of- function mutation of *Ttbk2* leads to spinocerebellar ataxia 11 [26] and neuron-specific deletion of *Ttbk2* induces loss of Purkinje cells [36], development of TTBK1-specific inhibitor such as ASO-*Ttbk1*, is necessary to avoid unwanted off-target effect. We found that in vivo administration of ASO-*Ttbk1* significantly suppressed pS396, pT231 and pT181 in oligomer form tau fraction in the hippocampal tissue in PS19 tau mouse. Our results support the finding that TTBK1 was the top kinase of pT231 in the unbiased analysis of tau phosphorylation in AD patients [10]. pS422 post-translational modification is found in pre-tangle formation in neurons with early-stage AD patients [37]. pS422 positivity in neurons are strongly correlated with cognitive decline and disease pathology than mature neurofibrillary tangle formation [38]. Furthermore, active immunization of tau-overexpressing THY-22 mice with the pS422 site reduced phosphorylation of tau in the insoluble fraction and improved spatial learning [39]. As predicted by the previous report showing co-expression of pS422 and TTBK1 protein in pre-tangles [5], our study showed that ASO-*Ttbk1* suppressed the level of pS422, which was significantly associated with TTBK1 protein level in the hippocampal tissue in PS19 mice. Furthermore, ASO-*Ttbk1* significantly reduced the level of pS396, an indicator of late-stage aggregation, in the P2 fibrillar form tau fraction. These results indicate that selective suppression of *Ttbk1* reduced the progression of tau phosphorylation and aggregation as determined by the phosphor-tau epitopes relevant to AD in PS19 mice.

The bulk RNA-seq dataset indicate that administration of ASO-*Ttbk1* induces GPCR and innate immune signaling including complement system, antigen presentation, interferon-γ pathways and enrichment of microglial activation markers. A recent study suggests that TTBK1 has functions outside of kinase domain such as microtubule and cytoskeleton organization or vesicle transport as a downstream pathway of WNT signaling [40]. Further investigation is warranted to investigate if loss of physiological TTBK1 function can lead to immune activation in PS19 mice.

Finally, our study has a few limitations. One limitation is that we use PS19 mice, a model of tauopathy, which express mutant human tau about fivefold higher than that of the endogenous mouse tau [16]. This prevents normal microtubule binding and culminates in aberrant kinase phosphorylation. The effect of ASO-*Ttbk1* therefore could be different for example, with late onset AD patients who do not possess mutation in tau. It is of our interest to investigate the effect of ASO-*Ttbk1* on different tauopathy models. Another limitation is that the strong effect of ASO-*Ttbk1* to prevent tau phosphorylation was observed only with ASO-*Ttbk1*#1 but not #2, likely reflecting their different efficacies in *Ttbk1* RNA knockdown. It is extremely important to test different ASO-*Ttbk1* candidates to validate our primary findings in future experiments.

In conclusion, these findings demonstrate that ASO-based approach successfully downregulates *Ttbk1* without affecting *Ttbk2* and suppresses tau phosphorylation in PS19 mice.

This study presents new possibilities to develop therapeutics for tauopathy-related neurodegenerative disorders.

### Supplementary Information

**Additional file 1: Figure S1.** Sequential fractionation from the mouse hippocampus. **Figure S2.** Whole gel images of immunoblotting. **Figure S3.** RNA-seq analysis of the hippocampal tissues from PS19 mice treated with ASO-*Ttbk1*. **Figure S4.** g:PROFILER analysis of DEG from the RNA-seq dataset of hippocampal tissues from PS19 mice treated with ASO-*Ttbk1#1*. **Figure S5.** GSEA analysis of the RNA-seq dataset of hippocampal tissues from PS19 mice treated with ASO-*Ttbk1*. **Figure S6.** qPCR analysis of the temporal cortex of PS19 mouse treated with ASO-*Ttbk1*. **Figure S7.** R session information.

**Additional file 2: Table S1.** Correlation of tau-phosphorylation and TTBK1 expression in the hippocampus. **Table S2.** Enriched biological pathways by GSEA analysis. **Table S3.** Enricher analysis of cell type enrichment.

## Data Availability

The datasets used and/or analyzed during the current study are available from the corresponding author on reasonable request.
