# Supplementary material for: Antisense oligonucleotide-based targeting of Tau-tubulin kinase 1 prevents hippocampal accumulation of phosphorylated tau in PS19 tauopathy mice
Source: Acta Neuropathol Commun. 2023 Oct 19;11:166. doi: 10.1186/s40478-023-01661-3 (PMC10585748; doi:10.1186/s40478-023-01661-3)
Supplement: Supplementary file 1 — Additional file 1: Figure S1. Sequential fractionation from the mouse hippocampus. Figure S2. Whole gel images of immunoblotting. Figure S3. RNA-seq analysis of the hippocampal tissues from PS19 mice treated with ASO-Ttbk1. Figure S4. g:PROFILER analysis of DEG from the RNA-seq dataset of hippocampal tissues from PS19 mice treated with ASO-Ttbk1#1. Figure S5. GSEA analysis of the RNA-seq dataset of hippocampal tissues from PS19 mice treated with ASO-Ttbk1. Figure S6. qPCR analysis of the temporal cortex of PS19 mouse treated with ASO-Ttbk1. Figure S7. R session information. [file 40478_2023_1661_MOESM1_ESM.pdf]

Supplemental Information

Antisense oligonucleotide-based targeting of Tau-tubulin kinase 1 prevents hippocampal accumulation of phosphorylated tau in PS19 tauopathy mice

Kayo Yukawa[1], Satomi Yamamoto-Mcguire[1], Louis Cafaro[1], Christine Hong[2], Fredrik Kamme[2], Tsuneya Ikezu[1,3*], and Seiko Ikezu[1,3*]

[1] Department of Pharmacology and Experimental Therapeutics, Boston University School of Medicine, Boston, MA 02118, USA

[2] Ionis Pharmaceuticals, Carlsbad, CA 92010, USA

[3] Department of Neuroscience, Mayo Clinic Florida, Jacksonville, FL 32224, USA

Contents

FIG. S1. Sequential fractionation from the mouse hippocampus

FIG. S2. Whole gel images of immunoblotting

FIG. S3. RNA-seq analysis of the hippocampal tissues from PS19 mice treated with ASO-*Ttbk1*

FIG. S4. g:PROFILER analysis of DEG from the RNA-seq dataset of hippocampal tissues from PS19 mice treated with ASO-*Ttbk1#1*

FIG. S5. GSEA analysis of the RNA-seq dataset of hippocampal tissues from PS19 mice treated with ASO-*Ttbk1*

FIG. S6. qPCR analysis of the temporal cortex of PS19 mouse treated with ASO-*Ttbk1*

FIG. S7. R session information

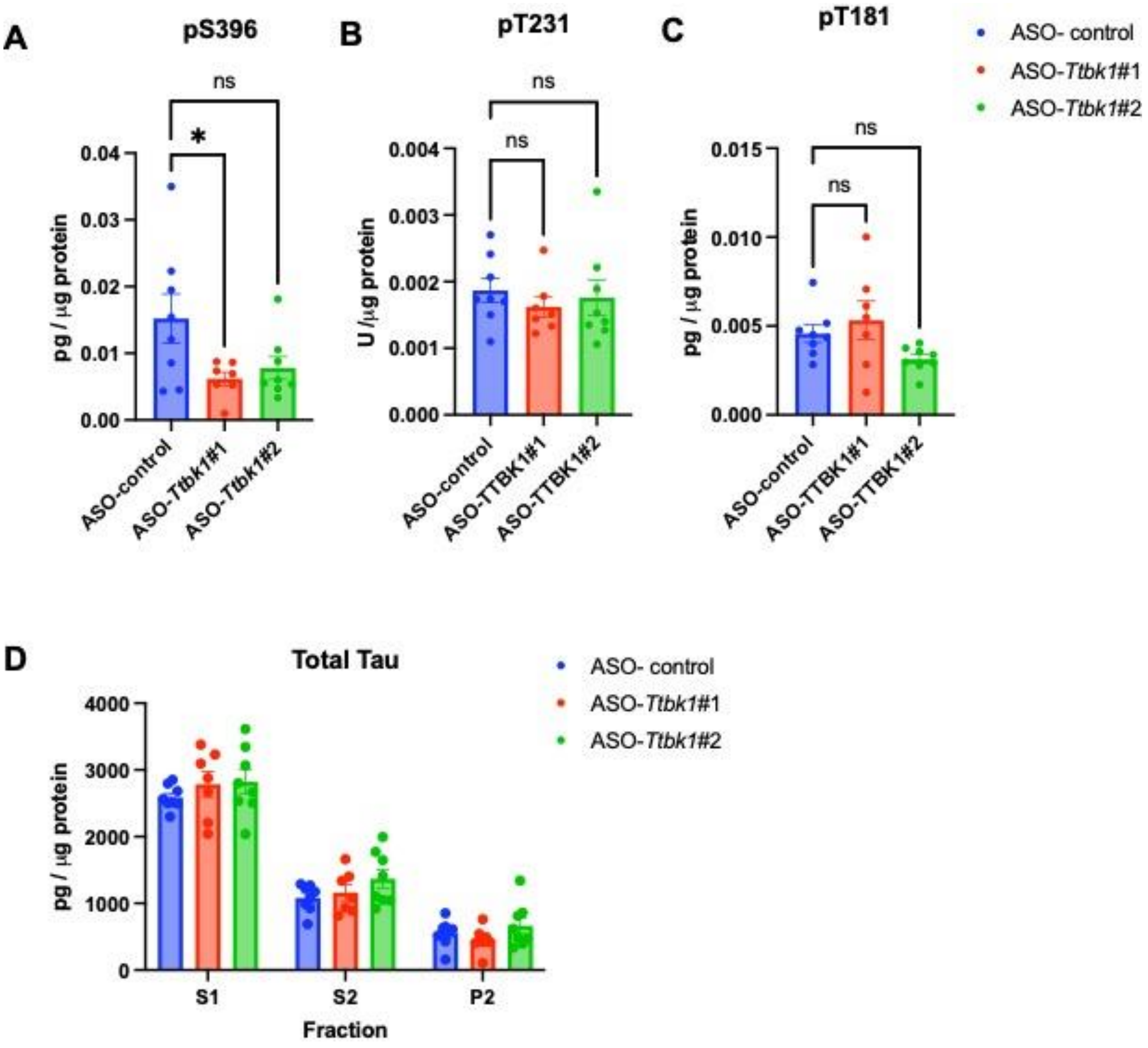

Fig. S1. Phosphorylated tau level in soluble fractions in the hippocampal tissues isolated from PS19 mice treated with ASO-*Ttbk1*

(A)-(C) ELISA-based quantification of p-tau for epitopes pS396, pT231, and pT181 for the TBS soluble (S1) fractions isolated from the hippocampal brain tissue in PS19 mice after ASO-*Ttbk1* or ASO-control treatment. *p <0.05 vs. ASO-control as determined by one-way ANOVA and Dunnett's multiple comparison. (ASO-control n=7; ASO-*Ttbk1* #1 n=6; ASO-*Ttbk1* #2 n=8 mice/group). (D) Total tau protein concentration in all fractions (S1: TBS soluble fraction, S2: sarkosyl-soluble fraction, P2: sarkosyl-insoluble fraction). Blue: ASO-control group, red: ASO-*Ttbk1*#1 group, and green: ASO-*Ttbk1*#2 group.

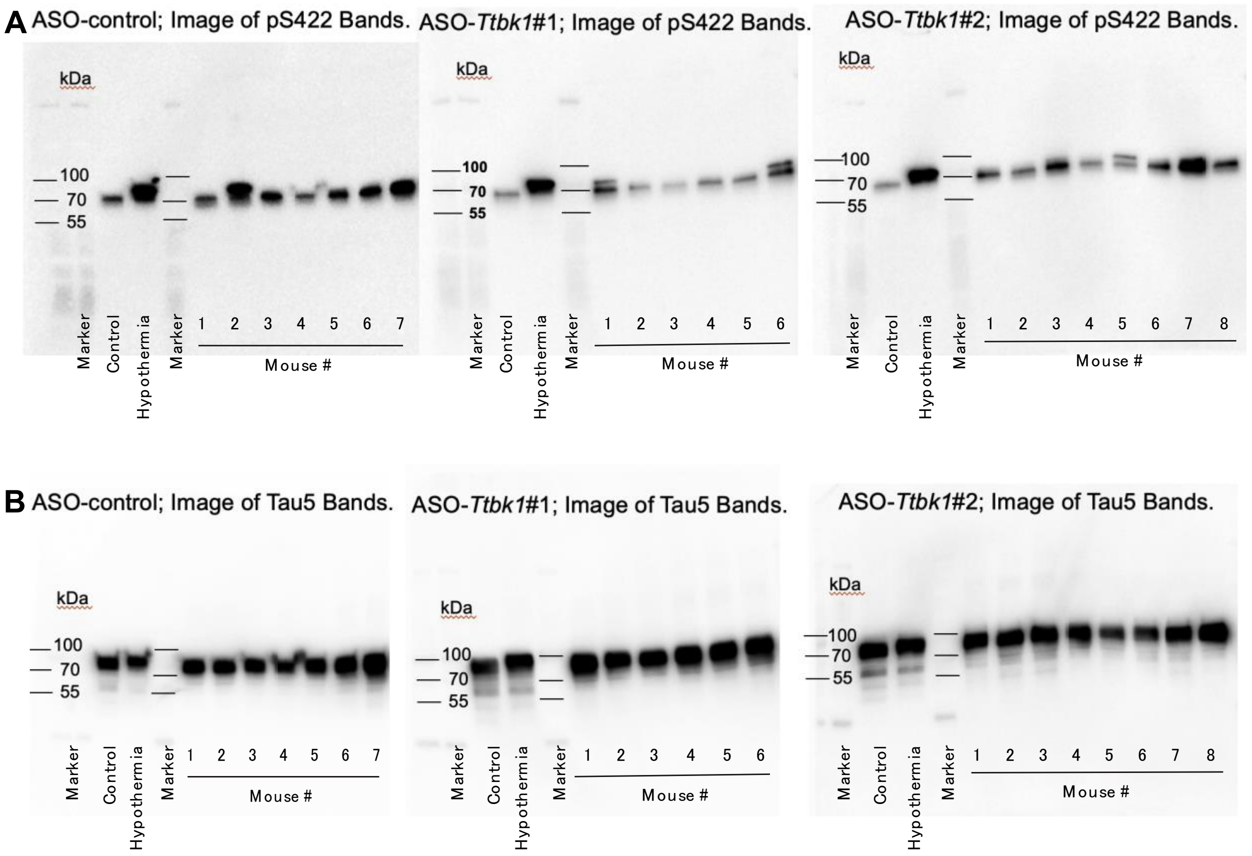

Fig. S2. Whole gel images of WB.

Western blot results against pS422 and total tau (tau5) antibodies with soluble fraction S1 isolated from the hippocampal tissue 8 weeks after the injection of ASO-*Ttbk1* or -control. C: WT mouse sample H: WT mouse samples under hypothermia to induce tau phosphorylation as a positive control, M: Marker, 1-8: Samples from PS19 mice injected with ASO-control, ASO-*Ttbk1*#1, or ASO-*Ttbk1*#2. （A） Immunoblotting image using anti-phospho-Tau (Ser422) polyclonal antibody (Invitrogen, #44-764G, Host: Rabbit), (B) Immunoblotting image using anti-Tau monoclonal antibody (TAU-5, Abcam, #ab80579, Host: Mouse).

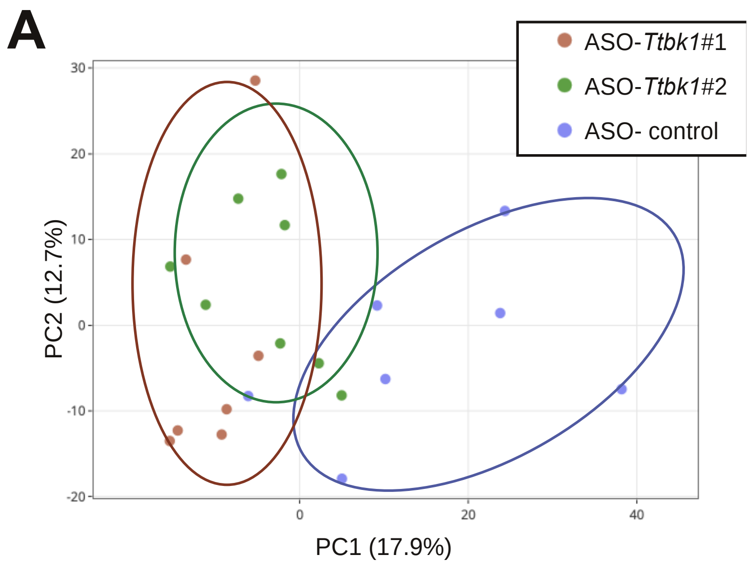

Fig. S3. Principal component analysis (PCA) of the RNA-seq data of the hippocampal tissues isolated from PS19 mice treated with ASO-*Ttbk1 or* ASO-*control*

ASO-*Ttbk1* treated groups (#1 and #2) were separated from ASO-control group in PC1 (17.9%) but not in PC2 (12.7%). ASO-control (n=7, blue), ASO-*Ttbk1*#1 (n=7, red), and ASO-*Ttbk1*#2 (n=8, green).

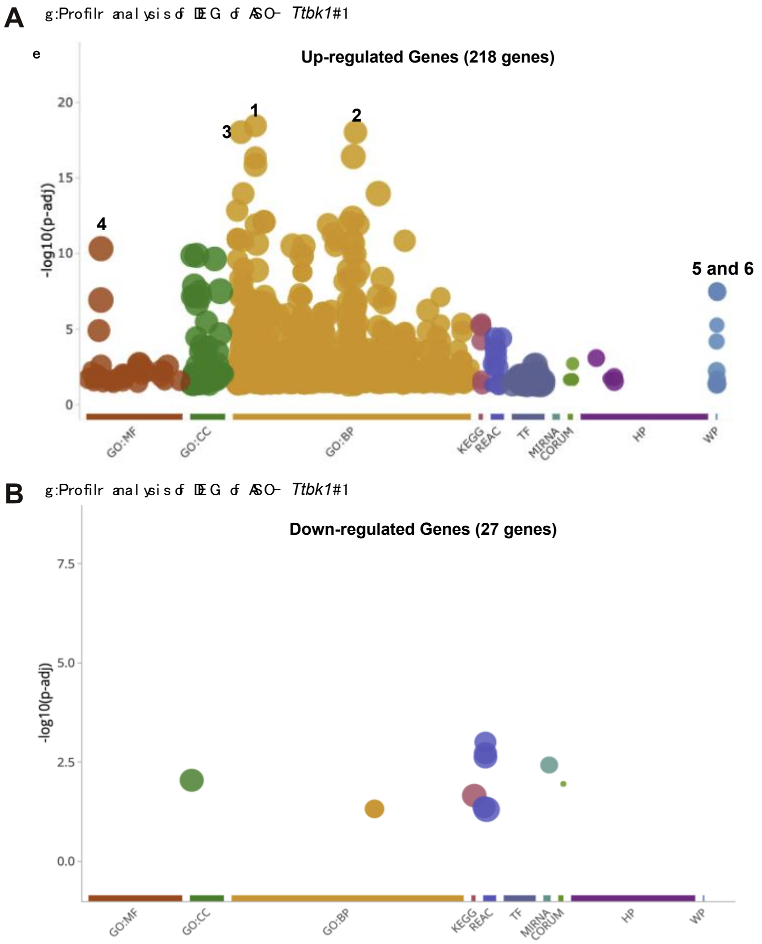

C

No.	Source	Term name	Term id	Term size	Adjusted p_value
1	GO:BP	immune response	GO:0006955	1851	3.32E-19
2	GO:BP	regulation of multicellular organismal process	GO:0051239	2933	3.11E-18
3	GO:BP	defense response	GO:0006952	1788	8.90E-18
4	GO:MF	protein binding	GO:0005515	10491	8.23E-11
5	WP	Microglia pathogen phagocytosis pathway	WP:WP3626	41	3.07E-08
6	WP	Tyrobp causal network in microglia	WP:WP3625	58	7.36E-08

Fig. S4. g:Profiler analysis of DEG in hippocampal tissues in PS19 mice treated with ASO-*Ttbk1*#1.

Manhattan plots of g;Profiler enrichment analysis showing up-regulated genes (A) and down regulated genes (B) by ASO-*Ttbk1* treatment. The X-axis represents the functional terms with color-code and data source. The data source is based on gene ontology (GO) for molecular function (MF), biological process (BP), and cellular component (CC), biological pathways (KEGG), Reactome (REAC), WikiPathways (WP), regulatory motifs in DNA (TF and MIRNA), protein databases (HPA and CORUM), and Human Phenotype Ontology (HP). Each circle represents a description of a group of genes in the category. The Y-axis represents -log10 (adjusted p-value). (C) The list of most significant pathways as determined by GO and WP. The

number (No.) indicates the circle annotated in the Manhattan plot (A) as the most significant pathway in each category.

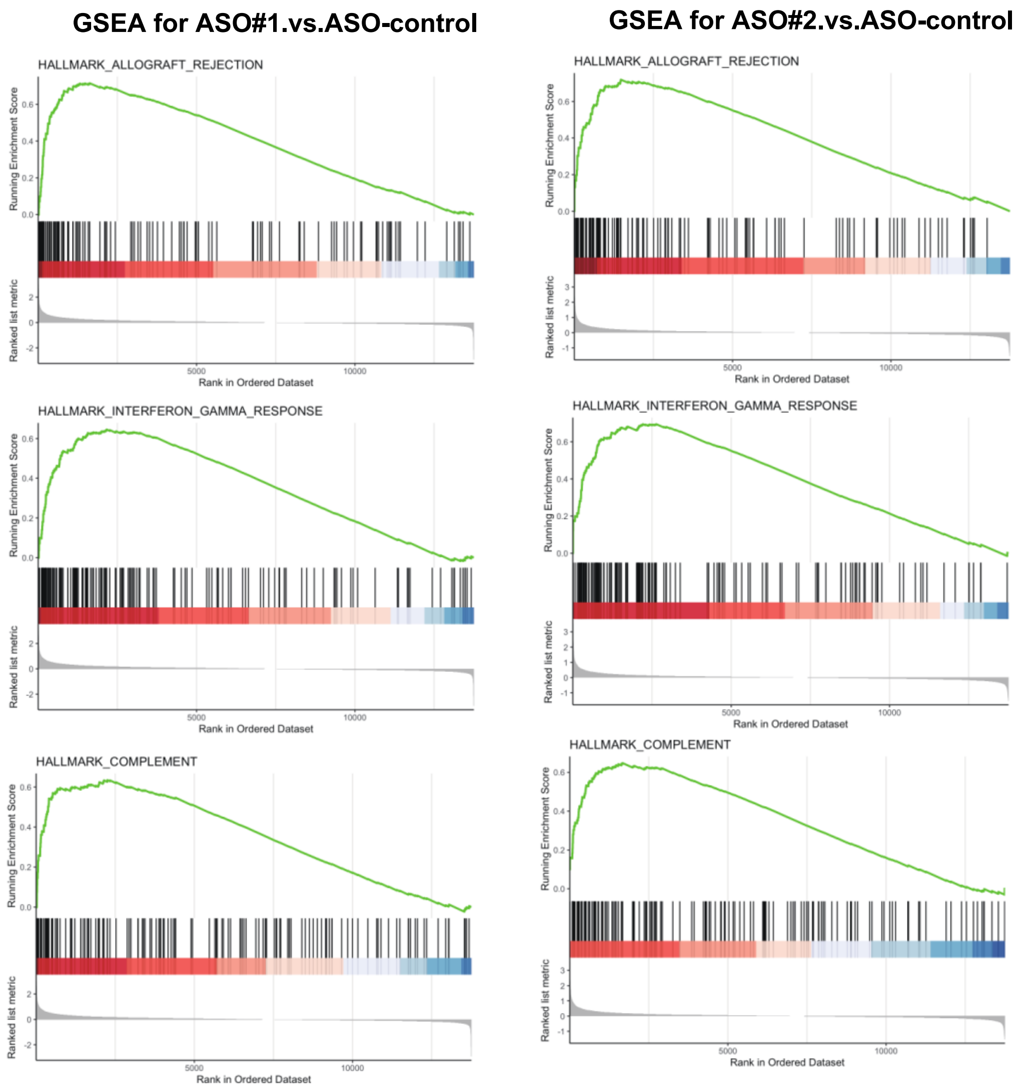

Fig. S5. GSEA analysis of RNA-seq data set with ASO-Ttbk1 treated PS19 mice. Commonly found Gene set enrichment analysis (GSEA) profile for ASO-Ttbk1#1 vs. ASO-control or ASO-Ttbk1#2 vs. ASO-control. The commonly enriched hallmarks are allograft rejection (top), interferon gamma response (middle), and complement pathways (bottom).

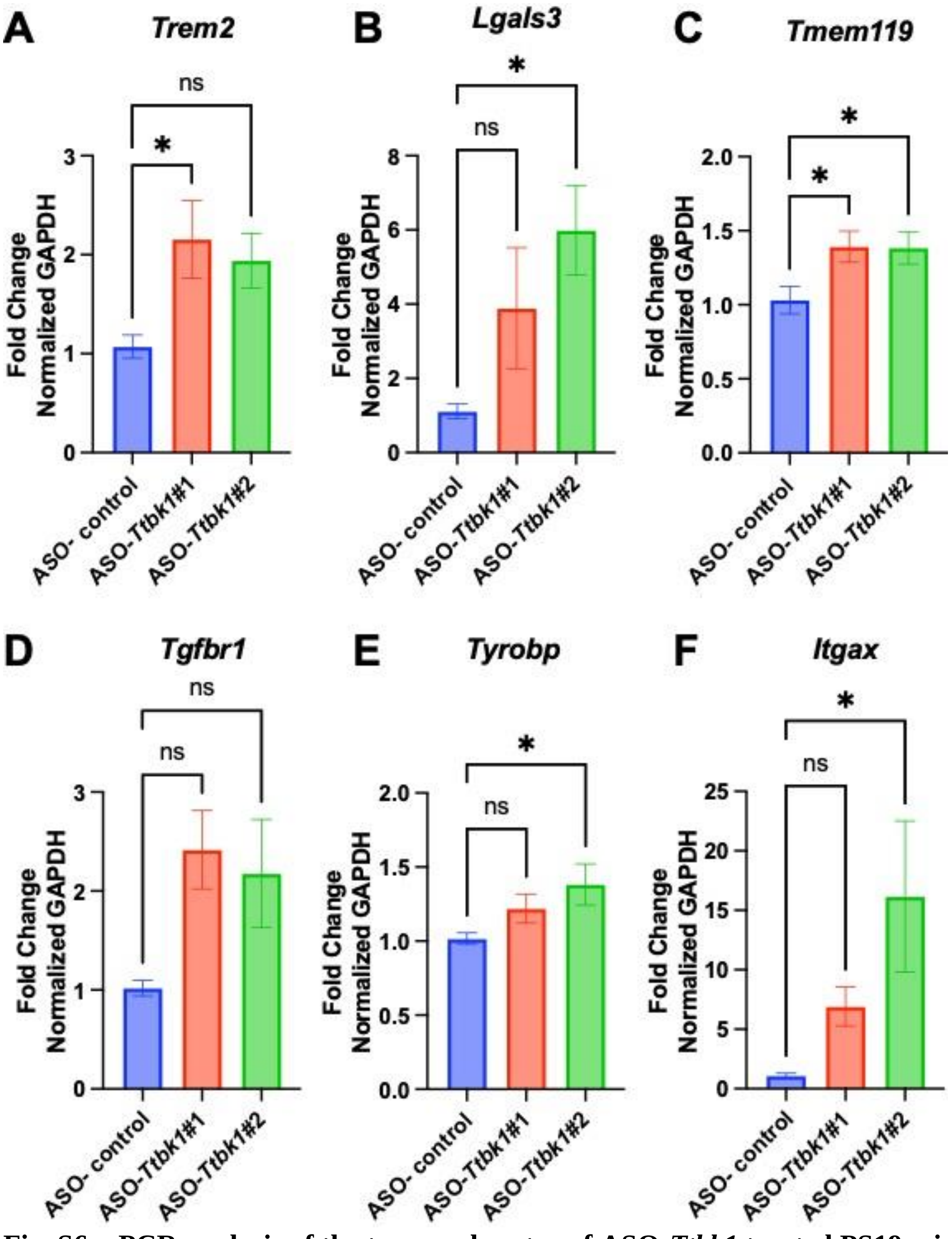

Fig. S6. qPCR analysis of the temporal cortex of ASO-*Ttbk1* treated PS19 mice
mRNA expression level of selected microglial genes was analyzed in the temporal cortex tissue in PS19 mice treated with ASO-*Ttbk1* #1 and #2 by qPCR, including (A) *Trem2*, (B) *Lgals3*, (C) *Tmem119*, (D) *Tgfbr1*, (E) *Tyrobp*, and (F) *Itgax*. All data were normalized to GAPDH, and the fold change relative to the expression level of the genes with ASO-control treated samples was presented. *p <0.05 vs. ASO-control treated group as determined by one-way ANOVA and Dunnett's multiple comparison. (ASO-control n=7, ASO-*Ttbk1* #1 n=6, ASO-*Ttbk1* #2 n=8 mice/group).

Fig. S7. R Session Information

```
## R version 4.0.2 (2020-06-22)

## Platform: x86_64-apple-darwin17.0 (64-bit)

## Running under: macOS  10.16

##

## Matrix products: default

## BLAS:   /Library/Frameworks/R.framework/Versions/4.0/Resource
s/lib/libRblas.dylib

## LAPACK: /Library/Frameworks/R.framework/Versions/4.0/Resource
s/lib/libRlapack.dylib

##

## locale:

## [1] en_US.UTF-8/en_US.UTF-8/en_US.UTF-8/C/en_US.UTF-8/en_US.U
TF-8

##

## attached base packages:

##  [1] parallel  stats4    grid      stats     graphics  grDevi
ces utils

##  [8] datasets  methods   base
```

```
##
## other attached packages:
##  [1] cowplot_1.0.0               gprofiler2_0.2.0
##  [3] GSVA_1.36.2                 GSEABase_1.50.1
##  [5] graph_1.66.0                annotate_1.66.0
##  [7] XML_3.99-0.5                gplots_3.0.4
##  [9] EnhancedVolcano_1.6.0       ggrepel_0.8.2
## [11] plotly_4.9.2.1              gt_0.2.2
## [13] DT_0.15                     SummarizedExperiment_1.18.1
## [15] DelayedArray_0.14.0         matrixStats_0.56.0
## [17] GenomicRanges_1.40.0        GenomeInfoDb_1.24.0
## [19] future.apply_1.6.0          future_1.18.0
## [21] BUSpaRse_1.2.2              enrichplot_1.8.1
## [23] msigdbr_7.1.1               org.Hs.eg.db_3.11.4
## [25] AnnotationDbi_1.50.0        IRanges_2.22.1
## [27] S4Vectors_0.26.0            Biobase_2.48.0
## [29] BiocGenerics_0.34.0         clusterProfiler_3.16.0
## [31] corrplot_0.84               circlize_0.4.10
## [33] viridis_0.5.1               viridisLite_0.3.0
```

```
## [35] RColorBrewer_1.1-2          clusterSim_0.49-1

## [37] MASS_7.3-52                 cluster_2.1.0

## [39] gridExtra_2.3               png_0.1-7

## [41] ComplexHeatmap_2.4.1        UpSetR_1.4.0

## [43] fgsea_1.14.0                edgeR_3.30.0

## [45] limma_3.44.1                tximport_1.16.0

## [47] openxlsx_4.1.5              writexl_1.3.1

## [49] readxl_1.3.1                forcats_0.5.0

## [51] stringr_1.4.0               dplyr_1.0.2

## [53] purrr_0.3.4                 readr_1.3.1

## [55] tidyr_1.1.2                 tibble_3.0.3

## [57] ggplot2_3.3.2               tidyverse_1.3.0

## [59] here_0.1                    knitr_1.30

## [61] tinytex_0.25                markdown_1.1

## [63] rmarkdown_2.3

##

## loaded via a namespace (and not attached):

##   [1] rappdirs_0.3.1           rtracklayer_1.48.0       bit64
_4.0.2
```

```
##   [4] data.table_1.13.0        RCurl_1.98-1.2           Annot
ationFilter_1.12.0
##   [7] generics_0.0.2           GenomicFeatures_1.40.0   RSQLi
te_2.2.0
##  [10] europepmc_0.4            bit_4.0.4                websh
ot_0.5.2
##  [13] xml2_1.3.2               lubridate_1.7.9          httpu
v_1.5.4
##  [16] assertthat_0.2.1         xfun_0.16                hms_
0.5.3
##  [19] evaluate_0.14            promises_1.1.1           fansi
_0.4.1
##  [22] progress_1.2.2           caTools_1.18.0           dbply
r_1.4.4
##  [25] igraph_1.2.5             DBI_1.1.0                htmlw
idgets_1.5.1
##  [28] ellipsis_0.3.1           crosstalk_1.1.0.1        backp
orts_1.1.9
##  [31] biomaRt_2.44.0           vctrs_0.3.2              ensem
bldb_2.12.0
```

```
##  [34] withr_2.2.0                 ggforce_0.3.2               trieb
eard_0.3.0
##  [37] BSgenome_1.56.0             GenomicAlignments_1.24.0 prett
yunits_1.1.1
##  [40] DOSE_3.14.0                 lazyeval_0.2.2              crayo
n_1.3.4
##  [43] labeling_0.3                pkgconfig_2.0.3             tween
r_1.0.1
##  [46] ProtGenerics_1.20.0         rlang_0.4.7                 globa
ls_0.12.5
##  [49] lifecycle_0.2.0             miniUI_0.1.1.1              downl
oader_0.4
##  [52] BiocFileCache_1.12.0        modelr_0.1.8                cellr
anger_1.1.0
##  [55] rprojroot_1.3-2             polyclip_1.10-0             Matri
x_1.2-18
##  [58] urltools_1.7.3              reprex_0.3.0                ggrid
ges_0.5.2
##  [61] GlobalOptions_0.1.2         rjson_0.2.20                bitop
s_1.0-6
```

```
##  [64] KernSmooth_2.23-17       Biostrings_2.56.0         blob_
1.2.1
##  [67] rgl_0.100.54             R2HTML_2.3.2              shape
_1.4.4
##  [70] qvalue_2.20.0            manipulateWidget_0.10.1  gridG
raphics_0.5-0
##  [73] scales_1.1.1             memoise_1.1.0             magri
ttr_1.5
##  [76] plyr_1.8.6               gdata_2.18.0              zlibb
ioc_1.34.0
##  [79] compiler_4.0.2           scatterpie_0.1.4          clue_
0.3-57
##  [82] Rsamtools_2.4.0          cli_2.0.2                 ade4_
1.7-15
##  [85] XVector_0.28.0           listenv_0.8.0             tidys
elect_1.1.0
##  [88] stringi_1.4.6            yaml_2.2.1                GOSem
Sim_2.14.0
##  [91] askpass_1.1              locfit_1.5-9.4            fastm
atch_1.1-0
```

```
##  [94] tools_4.0.2                rstudioapi_0.11          plyra
nges_1.8.0

##  [97] farver_2.0.3               ggraph_2.0.3             diges
t_0.6.25

## [100] rvcheck_0.1.8              BiocManager_1.30.10      shiny
_1.5.0

## [103] Rcpp_1.0.5                 broom_0.7.0              later
_1.1.0.1

## [106] httr_1.4.2                 colorspace_1.4-1         rvest
_0.3.6

## [109] fs_1.5.0                   splines_4.0.2            graph
layouts_0.7.0

## [112] shinythemes_1.1.2          ggplotify_0.0.5          xtabl
e_1.8-4

## [115] jsonlite_1.7.0             tidygraph_1.2.0          zeall
ot_0.1.0

## [118] R6_2.4.1                   pillar_1.4.6             htmlt
ools_0.5.0

## [121] mime_0.9                   glue_1.4.2               fastm
ap_1.0.1
```

```
## [124] BiocParallel_1.22.0      class_7.3-17              codet
ools_0.2-16
## [127] lattice_0.20-41          curl_4.3                  gtool
s_3.8.2
## [130] zip_2.1.0                GO.db_3.11.0              opens
sl_1.4.2
## [133] munsell_0.5.0            e1071_1.7-3               DO.db
_2.9
## [136] GetoptLong_1.0.2         GenomeInfoDbData_1.2.3    haven
_2.3.1
## [139] reshape2_1.4.4           gtable_0.3.0
```
